# Head and Neck Melanoma

**DOI:** 10.5402/2012/948302

**Published:** 2012-03-26

**Authors:** R. Shashanka, B. R. Smitha

**Affiliations:** ^1^Department of General Surgery, Hassan Institute of Medical Sciences, Karnataka, Hassan 573201, India; ^2^Department of Oral Pathology & Microbiology, Sri Hasanamba Dental College & Hospital, Karnataka, Hassan 573201, India

## Abstract

The incidence of malignant melanoma appears to be increasing at an alarming rate throughout the world over the past 30–40 years and continues to increase in the United States, Canada, Australia, Asia, and Europe. The behavior of head and neck melanoma is aggressive, and it has an overall poorer prognosis than that of other skin sites. The authors review the published literature and text books, intending to give an overall picture of malignant melanomas of the head and neck and a special emphasis on treatment considerations with controversies in treatment including biopsy, radiation therapy, sentinel node biopsy, and nodal dissection.

## 1. Background

Melanoma is a malignancy of pigment-producing cells (melanocytes) located predominantly in the skin but also found in the eyes, ears, GI tract, leptomeninges, and oral and genital mucous membranes. The incidence of malignant melanoma appears to be increasing at an alarming rate throughout the world over the past 30–40 years and continues to increase in the United States, Canada, Australia, Asia, and Europe [1–3]. Melanoma accounts for 65% of all deaths from skin cancer, reflecting its lethal nature.

Most head andneck melanomas (70–90%) occur on the face [4], with the cheek the most common site [5]. Malignant melanomas of the neck (7%) and scalp (3%) are much less frequent [6, 7]. Melanomas of the external ear constitute 7% of the head and neck melanomas [8]. Although malignant melanomas of all cutaneous sites are slightly more common in women, melanomas of the head and neck are twice as common in men [7, 9]. Head and neck melanoma also tends to affect a slightly older aged group than melanomas in other sites [10]. Head and neck melanoma in children is a very rare and is usually associated with giant congenital nevus [11]. 

 Melanoma that does not originate in the skin is a very rare tumor and is considered as one of the most deadly of all human neoplasms [12, 13]. Primary mucosal melanomas represent only 1.7–3% of all primary melanomas [1, 14]. 

Generally, survival from head and neck melanoma is reported as 17% at 5 years and the 10-year survival rate is 5% [15]. We have reviewed the published reports and text-books, intending to give an overall picture of malignant melanomas of the head and neck. 

## 2. Pathophysiology

The sequence of events in which normal melanocytes transform into melanoma cells, referred to as melanoma genesis, is poorly understood. It likely involves a multistep process of progressive genetic mutations that (1) alter cell proliferation, differentiation, and death and (2) impact susceptibility to the carcinogenic effects of ultraviolet radiation [16]. Recent data suggest multiple pathways of melanoma pathogenesis, with melanomas in sun-protected skin (trunk) developing in association with a high nevus count and intermittent ultraviolet radiation as opposed to those developing on sun-exposed skin in patients with low nevus counts and chronic sun exposure [17, 18].

In a recent meta-analysis, Gandini et al, [19] found a possible positive correlation between an individual's number of nevi and the overall risk of melanoma. Other risk factors include fair complexion, excessive childhood sun exposure and blistering childhood sunburns, an increased number of common and dysplastic moles, a family history of melanoma, the presence of a changing mole or evolving lesion on the skin, and, importantly, older age [20, 21].

 Finally, genetics may play a role. Patients having at least one affected first-degree relative possess a higher likelihood of developing malignant melanoma. The CDKN2A (p16) chromosomal mutation is the most commonly isolated genetic culprit [22].

## 3. Diagnosis

A new or changing mole or blemish is the most common warning sign for melanoma. Variation in color and/or an increase in diameter, height, or asymmetry of borders of a pigmented lesion are noted by more than 80% of patients with melanoma at the time of diagnosis. Symptoms such as bleeding, itching, ulceration, and pain in a pigmented lesion are less common but warrant an evaluation.

 The American Cancer Society developed the ABCDEs to serve as a simple guideline of early melanoma warning signs.

- Asymmetry: half the lesion does not match the other half.
- Border irregularity: the edges are ragged, notched, or blurred.
- Color variegation: pigmentation is not uniform and may display shades of tan, brown, or black; white, reddish, or blue discoloration is of particular concern.
- Diameter: a diameter greater than 6 mm is a characteristic, although some melanomas may have smaller diameters; any growth in a nevus warrants an evaluation.
- Evolving: changes in the lesion over time are a characteristic; this factor is critical for nodular or amelanotic (nonpigmented) melanoma, which may not exhibit the classic criteria above [23].

More recent use of the “ugly duckling” warning sign, wherein skin examination is focused on recognition of a pigmented or clinically amelanotic lesion that simply looks different from the rest, may assist with detection of lesions that lack the classic ABCDE criteria (e.g., nodular, amelanotic, or desmoplastic melanomas) [24].

Experienced visual inspection is often the key to distinguishing a melanoma from other common benign pigmented skin lesions, such as lentigo simplex, junctional nevus, compound nevus, intradermal nevus, blue nevus, amalgam tattoo, Addison's disease, and heavy metal poisoning [22].

## 4. Types of Melanoma

The appearance and growth of melanoma differ depending on the morphologic type.  Table 1 shows the incidence and features of six types of melanoma [25].

Superficial spreading melanomas are the most common type. They are typified by an initial radial (spreading) growth phase with eventual development of a vertical growth phase. Superficial spreading melanoma is frequently associated with a nevus and often occurs in younger patients.

Nodular melanomas are the next most common type. These exhibit vertical growth from their onset.

Lentigo maligna melanomas are characterized by a prolonged radial growth phase. They tend to start as a slow growing, flat patch in sun-exposed areas (often the face and neck). They have a proclivity for the dermal-epidermal junction and tend to follow hair follicles.

Acral lentiginous melanomas are characteristically located on the palms or soles. Not all such lesions are acral lentiginous; however, they have a defined histologic appearance.

Desmoplastic melanomas can be seen in association with preexisting melanocytic lesions and can more frequently be amelanotic, making their diagnosis more difficult. They tend to be characterized by infrequent metastasis with a higher local recurrence rate, as well as more frequent perineural involvement.

Mucosal melanomas most frequently present in the nose and/or sinuses, followed by the oral cavity and nasopharynx. They are rare lesions but have a poor prognosis. Because of their development in hidden, clinically silent areas, diagnosis often occurs late, requiring more radical treatment and contributing to the poorer prognosis [5].

## 5. Laboratory Studies

The most important aspects of the initial workup for patients with melanoma are a careful history, review of systems, and physical examination.

- Sentinel lymph node biopsy (SLNB) is generally indicated for pathologic staging of the regional nodal basin(s) for primary tumors greater than or equal to 1 mm depth and when certain adverse histologic features (e.g., ulceration, high mitotic rate, and lymphovascular invasion) are present in thinner melanomas.
- Published data have shown that baseline and surveillance laboratory studies (e.g., lactate dehydrogenase [LDH] level, liver function tests), chest radiography (CXR), and other imaging studies (e.g., CT scanning, positron emission tomography [PET] scanning, bone scanning, and MRI) are not typically beneficial for stage I/II (cutaneous) melanoma patients without signs or symptoms of metastasis [26–28].
- A metastatic workup should be initiated if physical findings or symptoms suggest disease recurrence. Screening CT or PET may be considered if the patient has documented nodal metastasis based on results from the SLNB, although the yield is low (0.5–3.7%) in the setting of regional nodal micrometastasis and correlates with increasing tumor thickness, ulceration of the primary tumor, and/or large tumor burden in the sentinel lymph node(s) [29].

### 5.1. Procedures

The criterion standard for melanoma diagnosis is histopathologic examination of clinically suggestive skin or mucosal lesions. An excisional biopsy (or deep saucerization technique) with narrow margins is preferred when possible. In the case of lentigo maligna, a broad, paper-thin shave biopsy or multiple smaller biopsies may be the best techniques. The biopsy report should generally include the following:

- tumor thickness (Breslow depth);
- presence of ulceration;
- anatomic level of invasion (Clark level), no longer necessary as per 2010 AJCC staging [30];
- presence of mitoses, noted as 0 or 1 or more per millimeter squared;
- presence of regression (associated with lower rates of sentinel node positivity and improved disease-free survival) [31];
- lymphatic/vessel (lymphovascular) invasion or vascular involvement;
- host response (tumor-infiltrating lymphocytes).

Immunohistochemical staining for lineage (S-100, homatropine methylbromide 45 [HMB-45], melan-A/Mart-1) or proliferation markers (proliferating cell nuclear antigen, Ki67) may be helpful in some cases for histologic differentiation from melanoma simulators [5].

### 5.2. Histological Findings

Malignant cells often nest or cluster in groups in an organoid fashion; however, single cell can predominate. The melanoma cells have large nuclei, often with prominent nucleoli and show nuclear pseudoinclusions due to nuclear membrane irregularity. The abundant cytoplasm may be uniformly eosinophilic or optically clear. Occasionally, the cells become spindled or neurotized in areas. This finding is interpreted as a more aggressive feature, compared with the findings of round or polygonal cell varieties [22].

### 5.3. Staging

In 2009, the American Joint Committee on Cancer (AJCC) Melanoma Task Force (Table 2) revised the staging system for cutaneous melanomas based upon the results of their multi-institutional study of 17,600 patients [32, 33]. Staging adheres to the traditional tumor-node-metastasis (TNM) classification system. This system classifies melanomas on the basis of their local, regional, and distant characteristics, as follows:

- stage I and II: localized primary melanoma
- stage III: metastasis to single regional lymph node basin (with or without in-transit metastases)
- stage IV: distant metastatic disease [34].

Estimated 5-year overall survival (OS) in the staging Table is based on analysis of worldwide data encompassing nearly 60,000 patients in the 2008 AJCC Melanoma Staging Database [35].

### 5.4. Clark Levels

Two popular microstaging systems for melanoma are the Clark levels and the Breslow thickness classification. In the past, Breslow depth of invasion has been shown to have prognostic significance, as has Clark level of invasion. In the new system, Breslow depth plays a more vital role, while Clark level is deemphasized (relevant only for T1 lesions).

Other changes include the presence or absence of tumor ulceration, as well as tumor (T) thickness limits at 1.0-mm, 2.0-mm, and 4.0-mm depth for defining T stage. Stages I and II were confined to clinical staging, while stages III and IV used pathologic information from the nodes to define staging. Where the 1997 system used the size of nodal metastases to judge prognosis, new data have shown that the number of metastatic nodes is more relevant. The new system reflects that relevance by basing the N (nodal) stage on number of nodes involved like single or 2-3 or ≥4 metastatic nodes. In-transit metastases, which were grouped with the T staging in the 1997 system, are now included with the N staging. In general, in-transit metastases have been recognized to portend a poorer prognosis, which is reflected in the new system. Distant metastases are now grouped into one of three groups: M1a (including subcutaneous nodules/distant nodes), M1b (confined to lung metastases), and M1c (for all other visceral sites). Elevated lactate dehydrogenase (LDH) serum level also is associated with poor prognosis, and patients with distant metastases and increased LDH are stage M1c regardless of site of metastasis [25].

### 5.5. Evaluation and Treatment

Suspicious lesions are defined by the ABCD(E) criteria and the last criterion, which is a relatively recent addition, emphasizing monitoring of benign lesions to evaluate change over time [23]. For suspicious lesions, a full thickness biopsy is crucial for adequate diagnosis of lesion depth and invasion.

### 5.6. Biopsy

The prognosis and treatment of cutaneous melanoma depend greatly on the thickness of the lesion. Thus, the key to evaluation of suspected lesions focuses on obtaining a full-thickness biopsy. Excisional biopsy is the best choice for small lesions or for large lesions in cosmetically favorable locations. Excisional biopsy should extend down to the subcutaneous fat, with a small (2-3 mm) peripheral margin. Punch biopsy can be performed for large lesions or for lesions with a low suspicion of melanoma in a cosmetically unfavorable location. The biopsy should be performed at the highest or thickest point of the lesion.

Incisional biopsy is not recommended. Likewise, techniques that do not permit a full-thickness sample, such as shave or curette biopsy, are discouraged. Furthermore, pigmented lesions should not be definitively treated with laser therapy, electrocautery, or cryotherapy unless biopsy analysis proves them to be noncancerous [36].

 Some controversy exists in a number of areas. In terms of excision of the primary site, thin melanoma margins are generally accepted to be 1 cm. For melanomas greater than 2 mm in thickness, 2 cm margins are the norm. However, Krown and Chapman [37] noted that the ideal margin width for these deeper lesions has not been adequately studied. More information regarding current excision margin recommendations is reviewed on the subject by Lens [38]. In the head and neck regions, adequate margins may not be possible or advisable due to cosmetic or functional concerns.

Radiation therapy also has been controversial in the past due to the fact that the radiosensitivity of melanoma was at least considered questionable. This is changing as more recent data becomes available. Adjuvant radiation therapy for neck disease has been shown to be beneficial for patients with aggressive disease [39]. There are also data that support radiation treatment alone for regional disease control in head and neck melanoma, although no direct comparison of radiation versus surgical treatment has been studied [40].

### 5.7. Elective Dissection of the Lymph Nodes

For most solid tumors, including cutaneous malignant melanoma, the most powerful predictor of survival is the status of the regional lymph nodes. As the understanding of the tumor biology of malignant melanoma continues to evolve, the traditional role for lymphadenectomy in the evaluation of at-risk regional nodes has been challenged.

But according to some studies, using this technique adequate disease removal (e.g., clinically negative neck, nonfixed disease, and small nodes) is possible [41]. Radical neck dissections, unless indicated because of disease extent, should be avoided due to unnecessary morbidity. Because of the high risk of occult involved nodes, neck dissection should accompany parotidectomy for positive parotid disease [42].

The site of the primary lesion must be considered when neck dissection is planned in order to remove all intervening lymphatic drainage to the suspicious or positive node. The primary site must also be considered when one plans elective lymph node dissection (ELND) for clinically negative necks. For primary lesions involving the parietal or frontal scalp, temple, lateral forehead, lateral cheek, or ear, superficial parotidectomy in conjunction with neck dissection is appropriate because the parotid may harbor the primary echelon nodes [36].

The debate on neck dissection continues as therapeutic nodal dissection has not been definitively shown to have a survival benefit; it improves locoregional control [43]. Adding further complexity to this debate, data show that recurrence rates in the neck after neck dissection are higher than those in the axillary or inguinal areas [44].

Thus, until recently, a strong argument could be made for the “wait-and-see approach” to the clinically negative nodal basin because of the morbidity of dissection without evidence of a clear survival benefit. But recent study has shown that high-dose interferon (IFN) alfa-2b can be given as an adjuvant treatment for high-risk melanomas and indicated that elective nodal dissection should not be delayed until disease is clinically detectable. In this study, relapse-free survival and overall survival rates improved in patients treated with IFN alfa-2b versus controls subject [45].

### 5.8. Sentinel Lymph Node Biopsy in the Head and Neck

 The widespread use of sentinel lymph node (SLN) biopsy in the management of head and neck melanoma has been limited by several concerns. One is that the lymphatic drainage in the head and neck region is complex, with multiple primary channels and the potential for multiple SLN sites. Secondarily excision of these nodes can be technically challenging as small distances between sentinel nodes make detection and isolation difficult. Furthermore, approximately 25–30% of the sentinel nodes is found within the parotid gland, and concern of facial nerve injury has led many surgeons to advocate superficial parotidectomy over SLN biopsy. And lastly, the cooperation of experienced pathologists and nuclear medicine staff are essential to the success of the procedure [36]. 

SLNs were successfully localized in greater than 90% of cases, with the combined use of blue dye mapping and gamma probe lymphoscintigraphy. 5 studies demonstrated success rates of 95% or better. The rate of tumor-containing SLNs ranged from 11–17%. Additionally, reviews by Schmalbach et al. and Loree et al. describe accurately localizing intraparotid SLNs in at least 93% of cases [46, 47].

### 5.9. Medical Care

Numerous adjuvant therapies have been investigated for the treatment of localized cutaneous melanoma following complete surgical removal. No survival benefit has been demonstrated for adjuvant chemotherapy, nonspecific (passive) immunotherapy, radiation therapy, retinoid therapy, vitamin therapy, or biologic therapy [48]. Adjuvant interferon (IFN), alfa-2b, is the only adjuvant therapy approved by the US Food and Drug Administration for high-risk melanoma (currently defined as stages IIB, IIC, and III), which is associated with a 40–80% chance of relapse and death. Various experimental melanoma vaccines also show promise in the adjuvant setting [49]. Monoclonal antibodies are also considered as a second-line treatment for unresectable or metastatic melanoma.

### 5.10. Prognostic Factors

 The most important prognostic factors for cutaneous melanoma of the head and neck are as follows: increasing clark level of invasion, increasing tumor thickness, site that is scalp, greater than 1 mitosis per high power field, clinical ulceration, component of epitheloid cells, especially with pleomorphism, presence of microscopic satellites, lack of tumor infiltrating lymphocytes, and regional lymph node metastasis [5].

## 6. Conclusions

The incidence of melanoma of the head and neck has been increasing dramatically in the last several decades. Much of this change is related to increased sun exposure in the general population. Head and neck melanoma is a complex disease especially in its treatment considerations. Thus a more aggressive treatment is done when morbidity is not significantly increased. As in any other cancer, best opportunity for cure lies in early and aggressive treatment.

## Figures and Tables

**Table 1:** Characteristics and incidence of morphologic subtypes of melanoma.

Subtype	Incidence	Special features
Superficial spreading	75%	Flat during early phase, typically from preexisting nevus
Nodular	15%	Early vertical growth
Lentigo maligna	10%	Prolonged radial growth
Acral lentiginous	2–8%	Palms, soles, and nail beds
Desmoplastic	low	Associated with perineural invasion
Mucosal	2%	Poorer prognosis

**Table 2:** AJCC 2009 revised melanoma staging.

Stage	TNM staging	Stage	Pathologic staging	Histologic/clinical features
Stage 0	Tis N0 M0	0	Tis N0 M0	Melanoma in situ

Stage IA	T1a N0 M0	IA	T1a N0 M0	Melanomas ≤ 1 mm without ulceration and mitosis < 1/mm^2^

Stage IB	T1b N0 M0	IB	T1b N0 M0	Melanomas ≤ 1 mm with ulceration or mitosis ≥ 1/mm^2^
	T2a N0 M0		T2a N0 M0	Melanomas 1.01–2 mm without ulceration

Stage IIA	T2b N0 M0	IIA	T2b N0 M0	Melanomas 1.01–2 mm with ulceration
	T3a N0 M0		T3a N0 M0	Melanomas 2.01–4 mm without ulceration

Stage IIB	T3b N0 M0	IIB	T3b N0 M0	Melanomas 2.01–4 mm with ulceration
	T4a N0 M0		T4a N0 M0	Melanomas 4 mm without ulceration

Stage IIC	T4b N0 M0	IIC	T4b N0 M0	Melanomas 4 mm with ulceration

Stage III	Any T ≥ N1 M0	IIIA	T1-4a N1a M0	Single regional nodal micrometastasis, nonulcerated primary
			T1-4a N2a M0	2-3 regional nodal micrometastasis, nonulcerated primary
		IIIB	T1-4b N1a M0	Single regional nodal micrometastasis, ulcerated primary
			T1-4b N2a M0	2-3 regional nodal micrometastasis, nonulcerated primary
			T1-4a N1b M0	Single regional nodal macrometastasis, nonulcerated primary
			T1-4a N2b M0	2-3 macroscopic regional nodes, no ulceration of primary
			T1–4a N2c M0	2-3 nodes, in-transit met(s)* and/or satellite lesion(s) without metastatic lymph nodes
		IIIC	T1-4b N1b M0	Single regional nodal macrometastasis, ulceration of primary
			T1-4b N2b M0	2-3 macroscopic regional nodes, ulceration of primary
			T1-4b N2c M0	2-3 nodes, in-transit met(s)* and/or satellite lesion(s) without metastatic lymph nodes, ulceration of primary
			Any T N3 M0	4 or more metastatic nodes, or matted nodes, or in-transit met(s)/satellite (s) with metastatic nodes (s)

Stage IV	Any T Any N M1	IV	Any T Any N M1	Distant metastasis
				M1a: distant skin, subcutaneous, or nodal mets with normal LDH levels
				M1b: lung mets with normal LDH levels
				M1c: all other visceral metastases with normal LDH levels
				Distant metastasis with elevated LDH levels
